# Dental health service utilization among older adults receiving home care services in south-eastern Norway

**DOI:** 10.2340/aos.v85.46262

**Published:** 2026-06-11

**Authors:** Hero Ibrahim Hassan, Vibeke Ansteinsson, Marte-Mari Uhlen-Strand, Ragnhild Hellesø, Rasa Skudutyte-Rysstad, Ewa A. Sz. Hovden

**Affiliations:** a Department of Public Health and Interdisciplinary Health Sciences, Institute of Health and Society, University of Oslo, Oslo, Norway; b Oral Health Centre of Expertise in Eastern Norway, Oslo, Norway

**Keywords:** Oral health, dependent older adults, dental services utilization, entitlements in public dental health services

## Abstract

**Objective:**

To map dental health service utilization (DSU) and associated factors among older adults receiving home care services (HCS) in south-eastern Norway, using Andersen’s model.

**Materials and methods:**

In this cross-sectional study of older adults receiving HCS aged ≥ 65 years, data on oral health practices, general health, and predisposing, enabling, and need-based factors were collected via interviewer-administered questionnaire.

**Results:**

Of 116 participants (mean age 83, 53.6% female), 67.2% reported annual DSU, while 24.1% used dental health services only for acute problems. Although 95.6% were entitled to free dental care in the public dental service (PDS), 66 were aware of this, and 56.5% made use of their rights. Awareness of entitlements was associated with more frequent PDS use. A higher self-reported number of teeth and using private dental services were also linked to higher DSU.

**Conclusions:**

The present study showed underutilization of dental health services among older adults receiving HCS and unawareness of entitlement of their rights in PDS. However, higher self-reported number of teeth and use of private dental services were linked to more frequent DSU. To address underuse, the PDS should improve awareness of entitlements, enhance information delivery, and further investigate reasons for non-utilization.

## Introduction

Good oral health plays an important role in healthy aging among older adults and is a key factor in promoting overall public health [1, 2]. The World Dental Federation defines oral health as the ability to perform various craniofacial functions without pain, discomfort or disease [3]. Oral diseases are among the most common noncommunicable diseases, affecting around 3.5 billion people worldwide [4]. Regular utilization of dental health services has a preventive effect on oral diseases, as early detection enables the identification of high-risk individuals and contributes to improved oral health outcomes and reduced oral health impacts throughout life [5, 6].

The dental care system in Norway consists of a private dental service and a public dental service (PDS). Private dental services primarily serve the adult population, and are paid fully out of pocket, with certain exceptions covered by national reimbursement schemes. The PDS provides dental care to prioritized groups in accordance with the Dental Health Services Act, including children, young adults, people with special needs, and older adults dependent on healthcare from home care services (HCS) [7]. Due to demographic changes, many older adults are living longer, remain dentate, and many have chronic diseases that make them dependent on healthcare [8]. Dependent older adults can apply for HCS through their municipality [7]. Municipalities are responsible for organizing and delivering HCS, ensuring that older adults receive the services they need. Older adults receiving HCS are a heterogeneous group, varying in terms of health conditions and age. The Norwegian general legislation regulates care for the older adults, including the provisions of necessary care and services [9]. Individuals who have been receiving healthcare from HCS for at least 3 months, and at a frequency of at least once per week, are eligible for free dental care through the PDS [7]. Despite these entitlements, only one out of five older adults in HCS utilize this service [10], and reasons for non-utilization have not been investigated.

Previous studies have demonstrated that factors such as age, general health, oral health behaviors, and income influence dental health services utilization (DSU) [11, 12]. Several studies have applied Andersen’s behavioral model of healthcare utilization to examine patterns and determinants of DSU [13–15]. According to this model, the utilization of health services is influenced by several underlying components: predisposing factors, enabling resources, need-based factors, and health behaviors [16]. Sociodemographic characteristics such as age, sex, and education are considered as predisposing factors that influence a person’s likelihood of using healthcare services. The model shows that enabling factors, such as income and place of residence, can either facilitate or hinder DSU. Moreover, need-based factors include both clinically assessed and self-reported oral health status, as well as symptoms such as pain and discomfort. Personal oral health behaviors, such as smoking, frequency of tooth brushing, as well as general health variables such as level of illness, medication use, and dependence on healthcare services, are also associated with DSU [17]. Understanding these behavioral and health-related influences on DSU is increasingly important considering broader societal changes such as demographic change. Not only is the population aging, but a growing number of older adults are also retaining their natural teeth into old age [18, 19]. The number of remaining teeth and overall health status have both been reported to influence DSU [12]. Furthermore, age-related changes in general health and the accumulation of chronic conditions over time may increase individuals’ dependence on healthcare services [20]. Such changes can also negatively impact oral health and lead to a need for more frequent dental care in older adults. Utilization of dental health services is less frequent among dependent older adults compared to healthier individuals, as indicated by previous studies [21]. However, existing research on DSU in Norway has often focused on the general population [14, 22], with limited attention given to vulnerable subgroups. There is a lack of knowledge regarding the utilization patterns among older adults receiving HCS, despite their increased need for both medical and dental care.

The aim of the present study was to map DSU among older adults receiving HCS in south-eastern Norway, and the factors that influence DSU among this group, using Andersen’s behavioral model.

## Materials and methods

### Participants and setting

The study population consisted of older adults receiving services from HCS from four different municipalities, including both urban and rural areas in south-eastern Norway. The inclusion criteria were being HCS receivers, aged 65 years or older, and having the cognitive and physical ability to participate in an interview. In 2020, the number of older adults receiving services from HCS across the four municipalities ranged from 335 to 9.300. HCS personnel recruited the participants during home visits. Prior to recruitment, the research team held informational meetings and digital presentations for the HCS personnel in each municipality. These meetings provided an overview of the study, and the types of questions participants would be asked, enabling HCS personnel to inform potential participants accurately. Individuals who were not competent to provide consent or had limited knowledge of Norwegian, were excluded. The participants who agreed to participate were contacted by the research team via telephone to arrange a home interview. Two members of the research team, comprising dentists (*n* = 2), dental hygienists (*n* = 4), and researchers (*n* = 2), conducted home interviews using an interviewer-administered questionnaire. Prior to data collection, a preparatory meeting was held with all interviewers present. During this meeting, each item in the questionnaire was reviewed and discussed to ensure a shared understanding and administration of the questionnaire. The questions were asked verbally, and the answers were recorded on an iPad by the research team. Interview durations varied from 20 to 60 min.

Data were collected from December 2020 to January 2023; the collection period was prolonged due to repeated interruptions due to coronavirus disease 2019 (COVID-19) restrictions.

### Questionnaire

The questionnaire consisted of participants’ background information, self-reported information about their oral health status, and utilization of dental services**.** The questionnaire was pretested on two volunteers aged > 70 years living at home, who were not receiving services from HCS and did not participate in the study. After minor linguistic and structural adjustments based on the pretest, the questionnaire was ready for use.

#### Outcome

The main outcome was dental service utilization (DSU), assessed by a question regarding regular dental visits: Do you go to the dentist/dental hygienist regularly? Response options included: (1) once per year or more (yes, more than once per year or yes, every year) and (2) less than once per year (every second year, at longer intervals, or only for acute problems). The Norwegian Directorate of Health recommends that recall intervals for dental examinations should range between 12 and 24 months and be determined based on an individual risk assessment [23]. Given that the study population is characterized by considerable health challenges and a relatively high number of remaining natural teeth [24], the majority of participants can be classified as being at increased risk for oral health problems. A 12‑month recall interval was therefore considered the most appropriate for this population. Accordingly, DSU in the present study was assessed using a 12‑month interval. If participants answered that they used the services only for acute problems, they were subsequently asked open questions about the reason for not going regularly to a dentist/dental hygienist. They were also asked about the reason for their last visit to a dentist/ dental hygienist, with response alternatives: regular check-up, dental treatment, or acute event.

Information about predisposing, enabling, and need-based factors, personal oral hygiene practices, and general health variables was collected. Predisposing factors included sex, age, education and living situation. Age was collected as a continuous variable and categorized into 10-year intervals for statistical analysis (65–74, 75–84, and ≥ 85 years) to facilitate comparison with other studies. Participants’ highest completed level of education was collected and categorized as basic (primary school or lower), middle (secondary school/vocational training), and higher (college/university level), and their living situation was assessed by asking if the participant lived alone (yes/no). Enabling factors included residence (urban/rural), household income and types of dental health services. Information on household income was based on yearly income (before tax) and recorded as low (≤ 300,000 NOK), middle (301–450,000 NOK), and high (> 450,000 NOK). Participants were asked where they receive dental care, with response alternatives: PDS, private dental services, or both PDS and private. Individuals who responded that they used both PDS and private dental services (*n* = 3) and those who responded, ‘do not know’ (*n* = 6) were not included in the analyses. To assess whether the participants knew if they had entitlements in PDS, they were asked: Do you know whether you are entitled to dental care through PDS? (yes/no).

**Figure 1:**
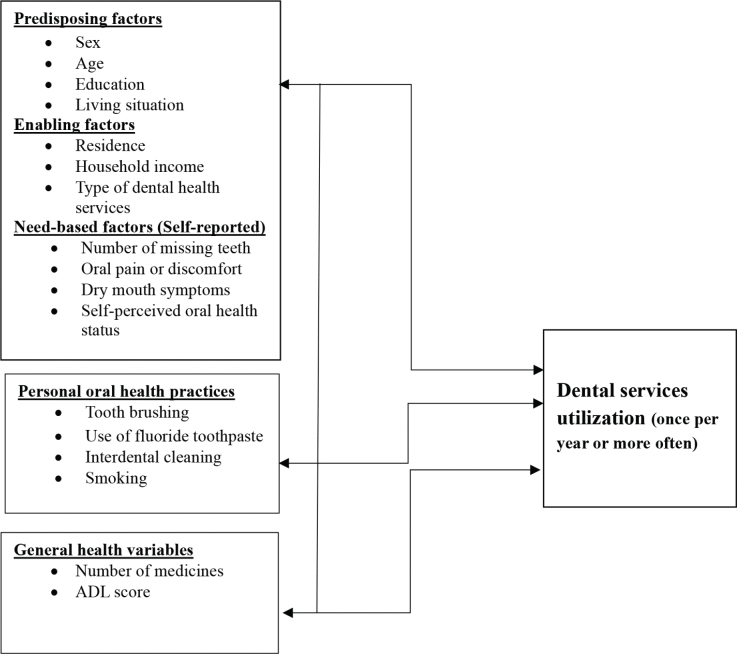
The dental services utilization model applied in the present study, adapted from Andersen [16].

Need-based factors included self-reported number of missing teeth, oral pain or discomfort, dry mouth symptoms and self-perceived oral health status. The DSU model for the present study is presented in Figure 1. Self-reported number of teeth was assessed by the question: How many natural teeth do you have? (response alternatives: all natural teeth, missing 1–4 teeth, missing 5–10 teeth, missing > 10 teeth, and no natural teeth left). The participants were asked if they had experienced oral pain or discomfort during the last month (yes/no). Information about self-reported dry mouth was obtained using the Summated Xerostomia Inventory-Dutch version (SXI-D) [24], and responses were dichotomized as yes/no. Participants were asked to rate their oral health as good, average, or poor. Personal oral health practices included frequency of tooth brushing (twice a day or more often/less than twice a day), daily use of fluoride toothpaste, interdental cleaning (yes/no), and cigarette smoking (yes/no).

The number of medicines used (0–4, 5–10, and > 10 medicines) and Activities of Daily Living (ADL) score served as general measures of health variables and were obtained from the patient journal system Gerica used by the HCS. ADL scores were dichotomized according to Norwegian national guidelines into scores ≤ 2 and > 2 [25]. A score > 2 indicates that the individual experiences some difficulties or is dependent on help from others to perform ADL.

Older adults receiving healthcare assistance from HCS once per week or more are entitled to the PDS. For this reason, information about frequency of services (< once per week or ≥ once per week) and the type of services provided by HCS (healthcare/home help/both) and duration (in years) these older adults received were collected.

### Ethics

Ethical approval for this study was obtained from the Regional Ethics Committee (REK 2020/ 32692) and the Norwegian Agency for Shared Services in Education and Research (Sikt, 540095). The study was supported by the Research Council of Norway (297462). All participants gave their written informed consent. The study is reported in accordance with the guidelines provided by ‘Strengthening the Reporting of Observational Studies in Epidemiology’ (STROBE) [26]^.^

### Statistical analysis

Nettskjema software, developed and operated by the University of Oslo (UiO), was used for designing the electronic questionnaire and for data collection. The platform Services for Sensitive Data (TSD) at UiO was used to store the data. Descriptive statistics in the form of frequency and percentage distributions were used to describe categorical variables. Nonparametric tests like Fisher exact test and Chi-square test were applied to explore associations between independent and dependent variables. All analyses were performed using Stata SE version 17 (Stata Corp. LLC version 17, Texas, USA), and the statistical significance level was set at α = 0.05.

## Results

Of the 116 older adults who were interviewed, 61 (52.6%) were female, and mean age was 83 years (standard deviation [SD]: 8.89, range: 65–102 years). Two-thirds (67.3%) of the respondents had middle or higher education. The majority lived alone. Thirty-six percent stated that they had a low income (Table 1). As shown in Table 2, half of the participants reported missing < 5 of their natural teeth, while oral pain and discomfort was reported by nearly one of six. Nearly 60% reported having good oral health. Two-thirds reported brushing their teeth twice per day or more frequently. The majority reported using fluoride toothpaste daily. Less than half reported use of interdental cleaning aids. Smoking cigarettes was reported by 11.2%. Data extracted from Gerica showed that the majority of the participants had an ADL sum score > 2, indicating a high level of dependency. Seventy percent used ≥ five medicines (Table 2). The results showed that the majority (95.6%) received healthcare from HCS once per week or more. Mean years of duration of HCS use was 7.46 (SD: 5.7) years.

**Table 1:** Distribution of participants according to predisposing and enabling factors, and association with dental health services utilization (≥ once per year) (n = 116).

Independent variables	*N* (%)	Dental health services utilization		*P*-values
		≥ Once per year *N* (%)	< Once per year *N* (%)	
Predisposing factors				
Sex				0.432
Female	61 (52.6)	43 (70.5)	18 (29.5)	
Male	55 (47.4)	35 (63.6)	20 (36.4)	
Age				0.081
65–74	23 (19.8)	13 (56.5)	10 (43.5)	
75–84	33 (28.5)	19 (57.6)	14 (42.4)	
≥ 85	60 (51.7)	46 (76.7)	14 (23.3)	
Education				0.894
Basic	33 (28.4)	23 (69.7)	10 (30.3)	
Middle	51 (44.0)	33 (64.7)	18 (35.3)	
Higher	27 (23.3)	18 (66.7)	9 (33.3)	
Unknown	5 (4.3)			
Living alone				0.292
Yes	91 (78.5)	59 (64.8)	32 (35 2)	
No	25 (21.5)	19 (76.0)	6 (24.0)	
Enabling factors				
Residence				0.886
Urban	53 (45.7)	36 (67.9)	17 (32.1)	
Rural	63 (54.3)	42 (66.7)	21 (33.3)	
Household income				0.420
Low	42 (36.2)	27 (64.3)	15 (35.7)	
Middle	27 (23.3)	19 (70.4)	8 (29.6)	
High	21 (18.1)	17 (81.0)	4 (19.0)	
Unknown	26 (22.4)			
Type of dental services				0.015
Public	39 (33.6)	23 (59.0)	16 (41.0)	
Privat	68 (58.6)	52 (76.5)	16 (23.5)	
Unknown	9 (7.8)			
Aware of having entitlement in PDS				0.880
Yes	66 (56.9)	44 (66.7)	22 (33.3)	
No	50 (43.1)	34 (68.0)	16 (32.0)	

*P*-values were calculated using Chi square test and Fisher’s exact test. The *N* in some cells is reduced because of missing values. Percentages are calculated column-wise.

**Table 2:** Distribution of participants according to need-based factors, personal oral health practices, and general health variables, and association with dental health services utilization (≥ once per year) (n = 116).

Independent variables	*N* (%)	Dental health services utilization		*P*-values
		≥ Once per year *N* (%)	< Once per year *N* (%)	
**Need-based factors**				
Self-reported number of missing teeth				**0.004**
Dentate, have all natural teeth	14 (12.0)	12 (85.7)	2 (14.3)	
Missing 1–4 teeth	44 (38.0)	30 (68.2)	14 (31.8)	
Missing 5–10 teeth	29 (25.0)	23 (79.3)	6 (20.7)	
Missing >10 teeth	21 (18.1)	12 (57.1)	9 (42.9)	
Edentulous	8 (6.9)	1 (12.5)	7 (87.5)	
Oral pain and discomfort				0.513
Yes	19 (16.4)	14 (73.7)	(5 (26.3)	
No	97 (83.6)	64 (66.0)	33 (34.0)	
Self-reported dry mouth				0.336
Yes	93 (82.3)	66 (71.0)	27 (29.0)	
No	20 (17.7)	12 (60.0)	8 (40.0)	
Self-perceived oral health				0.337
Good	71 (61.2)	47 (66.2)	24 (33.8)	
Average	35 (30.2)	26 (74.3)	9 (25.7)	
Poor	10 (8.6)	5 (50.0)	5 (50.0)	
**Personal oral health practices**				
Frequency of tooth brushing				0.513
Twice a day or more	78 (67.2)	54 (69.2)	24 (30.8)	
Less than twice a day	38 (32.8)	24 (63.2)	14 (36.8)	
Using fluoride toothpaste daily				0.522
Yes	101 (87.1)	69 (68.3)	32 (31.7)	
No	15 (12.9)	9 (60.0)	6 (40.0)	
Interdental cleaning daily				0.182
Yes	53 (45.7)	39 (73.6)	14 (26.4)	
No	63 (54.3)	39 (61.9)	24 (38.1)	
Cigarette smoking				1.000
Yes	13 (11.2)	9 (69.2)	4 (30.8)	
No	103 (88.8)	69 (67.0)	34 (33.0)	
**General health variables**				
Number of medicines				0.163
0–4	28 (24.1)	21 (75.0)	7 (25.0)	
5–10	56 (48.3)	33 (58.9)	23 (41.1)	
> 10	26 (22.4)	20 (76.9)	6 (23.1)	
Unknown	6 (5.2)			
ADL scores				0.514
Score ≤ 2	28 (24.1)	20 (71.4)	8 (28.6)	
Score > 2	85 (73.3)	55 (64.7)	30 (35.3)	
Unknown	3 (2.6)			

*P*-values were calculated using Chi square test and Fishers exact test. The *N* in some cells is reduced because of missing values. Percentages are calculated column-wise.

The number of participants reporting to visit a dentist or dental hygienist at least once every year was 78 (67.2%), while 28 (24.1%) reported seeking dental care only for acute problems (not shown in table). The most common reasons for not visiting the dentist were no need for dental treatment (46.4%) and general health issues, (27.0%) (not shown in Tables 1 and 2). Most of the participants (78.5%) reported that their last dental appointment was with a dentist. The reason for last dental/dental hygienist visit were reported to be a regular check-up by 51 participants (44%), while 29 (25%) indicated that their last visit was for dental treatment and 22 (19%) reported attending due to an acute problem (not shown in the Tables 1 and 2). More than half of the participants (56.9%) reported to have entitlements in the PDS. Only 35 individuals (56.5%) of them reported to have entitlement in PDS have reported utilizing PDS (not shown in the table). Those who were aware of their entitlements to the PDS used the services from the PDS more often than those who were not (*P* = 0.000). Overall, there were no significant associations between DSU and predisposing factors (Table 1). No statistically significant association was found between age and DSU, although descriptive data indicated higher DSU among individuals aged ≥84 years than the other age groups (Table 1).

With respect to enabling factors, participants who used private dental services utilized dental health services more regularly than those who used PDS (*P* = 0.015). Older adults with high income reported using dental health services more often than participants with low or medium income; however, this result was not statistically significant (Table 1).

Regarding need-based factors, statistically significant associations were found between self-reported number of teeth and DSU (*P* = 0.004) (Table 2). More frequent DSU was observed among participants reporting oral pain and discomfort, self-reported dry mouth, and good or average self-perceived oral health. However, these associations were not statistically significant. Additionally, for general health variables, less frequent DSU was reported by participants with ADL score ≥ 2 compared to those with ADL scores < 2 (non-significant). (Table 2).

## Discussion

This study mapped the utilization of dental health services among older adults receiving services from HCSs in south-eastern Norway. It also explored the association between predisposing factors, enabling factors, need-based factors, oral health practices, general health and DSU. To our knowledge, this is the first study to focus on DSU by older adults receiving HCS in Norway. The results showed underutilization of dental health services among older adults receiving HCS, and unawareness of entitlement of their rights in PDS. Awareness of entitlement to free dental care and use of private dental services were both associated with higher DSU. While the number of remaining natural teeth showed a significant association with utilization, age, income, ADL status, and general health variables were not statistically significant.

In the present study, nearly two-thirds of the participants utilized dental health services once or more often per year, which is a large proportion compared with studies conducted in other countries among dependent older adults [21, 27]. However, the proportion of older adults utilizing dental services in our study is lower when compared with DSU by the general population in Norway, which was 88% among adults aged ≥60 years [28]. A cohort study of the general population of older adults in both Norway and Sweden revealed that nearly 90% utilized dental services at least once annually [29]. Previous research suggests that, with increasing age, individuals may adjust their expectations regarding oral health and prioritize other aspects of life [30]. As part of adapting to age‑related changes, oral health problems may become less salient. This may help explain why 46.4% of participants reported no perceived need for dental treatment as a reason for not utilizing dental services regularly. Such perceptions may be shaped by factors including health literacy, cultural norms, previous dental experiences, and socioeconomic circumstances [31]. Previous research has shown that among older adults, self‑reported dental needs are consistently lower than clinically assessed needs [32], with many individuals therefore not seeking dental care. This underreporting may reflect not only limited health literacy and expectations, but also health‑related changes that influence sensory perception, functional capacity, and the recognition of oral health problems [33]. Another reason for the lower DSU among our study participants compared with the general population could be that the participants in our study are dependent on services from HCS. Additionally, a quarter of the participants in this study use dental services less than once per year or only for acute issues. Health problems were commonly reported as the reason for irregular dental service use. Participants with ADL score ≥ 2 reported DSU less frequently than participants with ADL score < 2. This finding is consistent with other studies from Europe and the United States, where general health issues were identified as a significant barrier to dental care utilization [12, 21]. A study from Belgium showed that dental attendance decreased with increasing frailty level among older adults [34]. Dependent older adults with poor general health tend to have poorer oral health than healthy adults [35], which should lead to higher utilization of dental services due to increased risk of oral disease [36, 37]. Recent research in Norway reported that some older adults use PDS primarily for acute issues, due to shortcomings in collaboration, communication, and lack of digital information exchange between HCS and PDS [38]. The organization of the HCS and PDS also plays an important role and warrants attention. In Norway, these services are managed at different administrative levels, with HCS under municipal administration and PDS under county administration. Recent research from Norway has highlighted challenges in the delivery of necessary services to older adults who receiving services from HCS, as reported by both HCS personnel and dental healthcare personnel [38, 39]. Since HCS personnel already have the responsibility for daily personal hygiene, oral health follow-up could be integrated into their routines. They can identify dental issues by conducting regular assessments of older adults’ dental health and providing informational resources. Collaboration with PDS can also ensure better access to dental care for older adults, and concrete strategies to implement such collaboration should be considered in future practice. Better collaboration would increase the awareness of entitlements the older adults have in PDS.

Our findings show that more than 40% of older adults who are aware of their entitlement to free dental care through the PDS still use private dental services. This raises important questions about accessibility, quality of care, and individual preferences. Understanding the factors that influence the utilization of dental health services among older adults is essential for improving healthcare strategies and ensuring optimal dental care for this vulnerable population. Dental health services are provided through private dental care for the majority of adults. However, when individuals become dependent on healthcare, and receiving services form HCS, they are entitled to receive dental care in the PDS. Older adults receiving HCS, who have established patient–dental relationships with private dentists, may be reluctant to transition to PDS, even after obtaining entitlements [40]. Many older adults continue with the same dentist because they value trust, comfort and continuity in care. Familiarity and positive past experiences play a key role in their choice [41]. Consequently, those who choose to continue receiving dental care from private dentists must cover the associated costs out of pocket, which may create financial barriers to accessing necessary dental care.

It has previously been reported that variables such as education levels and income have an impact on DSU [42, 43]. In our study, while the results from predisposing factors were not statistically significant, DSU was higher among individuals aged ≥ 84, and those with higher income compared to other age groups and participants with lower income. Similar results have been reported among the healthy general population in Norway [22, 29]. Participants who use private dental services utilize dental services more frequently than those individuals who use the PDS, and this is in line with previous research [14]. Given that financial constrains have historically limited the utilization of dental services [11, 43], it would be reasonable to expect an increase in use when such services are offered at no cost for dependent older adults in Norway. Speculatively, individual preferences and the possibility of continuing dental care with the same clinician could have played a role in selecting private care, but this needs to be further investigated.

Results in our study revealed a significant association between the number of teeth (need-based factors) and DSU. This result is in line with those in earlier studies [44]. However, it remains unclear whether this is due to particularly diligent dental care or an increased need for dental treatment. Additionally, the results indicated that individuals reporting pain, dry mouth, good or average oral health also reported more frequent DSU, but these findings were not significant. With the majority of participants having many of their own natural teeth, good oral hygiene is important. Maintaining good oral hygiene and emphasis on preventive oral care are particularly important in this group due to the high prevalence of dry mouth, which is a significant risk factor for poor oral health [24]. As such, it is concerning that over 30% of participants in this study exhibited inadequate oral hygiene practices.

Given the limited research on DSU among dependent older adults in Norway, the results from this study showing lower DSU among this group, and that one third do not make use of the services available from PDS, provide important insights. These findings indicate a need for improved information strategies regarding entitlement in PDS and the future exploration of the reason underlying non-use of available services. As the number of older adults with their own teeth who rely on help from HCS continues to grow, more extensive research in this area is essential. Both dental health services and HCS share the responsibility of ensuring that this group receives the rights and dental care to which they are entitled. Multilevel intervention and cooperation between these two services are necessary to deliver a good service to older adults receiving services from HCS and improve DSU among this group [45, 46].

### Limitations

This study had a cross-sectional design, and it is therefore not possible to establish causal associations from the results. Our results are based on self-reported data, which may introduce bias, as individuals reporting more positive behavior could be overrepresented [47]. However, research from Norway has demonstrated that self-reported oral health variables, such as number of teeth and edentulousness, are valid measures [48]. Data collection faced interruptions and delays due to COVID-19 restrictions, which reduced the number of participants. As the questionnaire was interviewer-administrated, the interviewers’ manner of asking the questions may have influenced the answers [49]. The possibility of selection bias cannot be completely ruled out as the participants in this study were recruited by HCS personnel, using convenience sampling. The possibility of selection bias related to health‑related challenges within the study population should be acknowledged. Prior evidence indicates that older adults with better health status and higher functional capacity are more likely to participate in research studies than those with poorer health [50]. As a result, the present sample may disproportionately represent relatively healthy and well‑functioning older adults compared to the overall population of HCS. Therefore, the external validity of the findings is limited, and generalizations should be made with caution. However, the sample distribution was comparable to national data from Statistics Norway for older adults receiving HCS in terms of sex, education, and income [51].

## Conclusion

The present study showed underutilization of dental health services among older adults receiving HCS and unawareness of entitlement of their rights in PDS. Having higher number of remaining teeth and using private dental services was significantly associated with higher DSU. Better cooperation between HCS and the PDS is necessary to raise awareness of and improve information about entitlements among older adults. Further research should explore reasons for non-utilization of the PDS among those entitled to them.

## Data Availability

The data that support the findings of this study are not publicly available because they contain information that could compromise the privacy of research participants. Further inquiries can be directed to the corresponding author (HIH).
